# Factors determining access to oral health services among children aged less than 12 years in Peru

**DOI:** 10.12688/f1000research.12474.1

**Published:** 2017-09-12

**Authors:** Diego Azañedo, Akram Hernández-Vásquez, Mixsi Casas-Bendezú, César Gutiérrez, Andrés A. Agudelo-Suárez, Sandra Cortés

**Affiliations:** 1 Center of Research on Population, Universidad Católica los Ángeles de Chimbote (ULADECH-Católica, Chimbote, 02800, Peru; 2 Universidad Privada del Norte, Lima, 15434, Peru; 3 Grupo de Modelamiento Matemático y Simulación Numérica, Universidad Nacional de Ingeniería, Lima, Peru; 4 Instituto Nacional de Estadística e Informática, Lima, 15072, Peru; 5 Medicine School, Universidad de Piura, Lima, Peru; 6 Center of Research and Extension, Facultad de Odontología, Universidad de Antioquia, Medellín, 050010, Colombia; 7 Advanced Center for Chronic Diseases (ACCDis), Santiago, 8330077, Chile; 8 Medicine School, Pontificia Universidad Católica de Chile, Santiago, 8330077, Chile

**Keywords:** Factors Associated; Oral Health; Access, Oral Health Services; Children; Peru

## Abstract

**Background: **Understanding problems of access to oral health services requires knowledge of factors that determine access. This study aimed to evaluate factors that determine access to oral health services among children aged <12 years in Peru between 2014 and 2015.

**Methods:** We performed a secondary data analysis of 71,614 Peruvian children aged <12 years and their caregivers. Data were obtained from the Survey on Demography and Family Health 2014-2015 (Encuesta Demográfica y de Salud Familiar - ENDES). Children’s access to oral health services within the previous 6 months was used as the dependent variable (i.e. Yes/No), and the Andersen and col model was used to select independent variables. Predisposing (e.g., language spoken by  tutor or guardian, wealth level, caregivers’ educational level, area of residence, natural region of residence, age, and sex) and enabling factors (e.g. type of health insurance) were considered. Descriptive statistics were calculated, and multivariate analysis was performed using generalized linear models (Poisson family).

**Results:** Of all the children, 51% were males, 56% were aged <5 years, and 62.6% lived in urban areas. The most common type of health insurance was Integral Health Insurance (57.8%), and most respondents were in the first quintile of wealth (31.6%). Regarding caregivers, the most common educational level was high school (43.02%) and the most frequently spoken language was Spanish (88.4%). Univariate analysis revealed that all variables, except sex and primary educational level, were statistically significant. After adjustment, sex, area of residence, and language were insignificant, whereas the remaining variables were statistically significant.

**Conclusions: **Wealth index, caregivers’ education level, natural region of residence, age, and type of health insurance are factors that determine access to oral health services among children aged <12 years in Peru. These factors should be considered when devising strategies to mitigate against inequities in access to oral health services.

## Introduction

According to the World Health Organization, dental caries is a prevalent chronic disease, with an estimated 60%–90% of school-age children and almost all adults affected in 2012 ( https://goo.gl/D8sh9n). Caries and other oral diseases may negatively affect the health and quality of life of people, particularly children ^1– 3^. Failing to treat caries might cause tooth loss that can significantly alter chewing, phonation, and occlusion ^2^. Likewise, the presence of caries during childhood can predict the development of caries in adulthood ^4^. High quality and timely preventive dental interventions can reduce the occurrence of caries and avoid complications in later life. However, no recent nationwide studies have analyzed the prevalence of dental caries in Peru.

A national study conducted by the Ministry of Health of Peru in 2001–2002 ( https://goo.gl/Zppe36) reported that the occurrence of dental caries was 90.4% of children aged 6–15 years, with 34.4% being diagnosed with dental conditions, such as dental pain or evident dental or periodontal infection, requiring urgent attention. Another study ^5^ conducted on the Social Security Health Insurance in 2013 showed that the prevalence of caries was 79.8% and 90.4% of children aged 3–5 and 6–12 years, respectively. Although no updated nationwide data regarding the prevalence rates of dental caries are available, studies ^6, 7^ indicate that the rates remain high (75.9% for children aged 6–7 years and 91.2% for children aged 11–12 years). Thus, there is a need to evaluate and treat dental conditions in Peruvian children and improve the promotion and prevention of oral diseases. Despite this need to improve provision, it should be acknowledged that Peru is a developing country with limitations such as large socioeconomic gaps, fragmented healthcare systems ^8^, limited provision of dental services at health centers, and few professional odontologists ^9– 11^. Moreover, cultural factors can affect children’s access to dental services, including their parents’ education and/or knowledge ^12^. Although these factors determine access to oral health services, most factors vary by region, country, and in response to other factors ^9^.

To tackle the problem of poor access to oral health services in Peru, a better understanding of the factors that determine access is required. This knowledge can be used to develop strategies to improve health inequalities and care provision ^9, 13^. Over the recent years, the Survey on Demography and Family Health (Encuesta Demográfica y de Salud Familiar - ENDES) has begun to include data related to oral health. Although its results have been descriptively reported nationwide, no existing studies have assessed whether any of the identified factors positively or negatively influence the oral health of Peruvian children.

We used the latest oral health data from ENDES (2014–2015) to evaluate factors that determine access to oral health services among children aged <12 years in Peru and to provide current data to empower policy makers when developing strategies to improve oral health in Peru.

## Methods

### Study design and information sources

We performed a secondary analysis of data obtained from ENDES 2014 and 2015 ( Dataset 1), which was performed by the National Institute of Statistics and Informatics of Peru (Instituto Nacional de Estadística e Informática - INEI). This cross-sectional survey involved self-weighted, stratified, two-stage, and independent probabilistic sampling with nationwide, regional, and urban/rural representation, in accordance with INEI specifications ( https://goo.gl/2AvdZ8) ( https://goo.gl/jE8Wt3). ENDES aimed to update our knowledge of the demographic and health statuses of mothers or caregivers and their children aged <5 years. Other target populations were fertile women (those aged 15–49 years) and all male and female children aged <12 years ( https://goo.gl/87q8mT).

ENDES 2014–2015 The data was obtained from Instituto Nacional de Estadística e Informática ( http://iinei.inei.gob.pe/microdatos/) and merged by the authors. Click here for additional data file. Copyright: © 2017 Azañedo D et al. 2017 Data associated with the article are available under the terms of the Creative Commons Zero "No rights reserved" data waiver (CC0 1.0 Public domain dedication).

### Dependent variable

A dependent variable was built to categorize minors into those who had accessed oral health services within the previous 6 months and those who had either accessed services at >6 months ago or those who had no access. Thus, access to an oral health service within the previous 6 months (yes/no) was determined to be the dependent variable.

### Independent variables

Independent variables were contextualized by considering Andersen and col. model of access ^14^ on the basis of predisposing, enabling, and need factors. This model has been widely used for evaluating factors that determine access to health services ^15^. However, we could not include any health need factors because ENDES does not have any suitable variables or proxies for the measurement of the factors.

***Predisposing factors.*** We included the following variables as predisposing factors: language spoken by the caregiver (i.e., Spanish or any other language), quintil of wealth, caregivers’ educational level, area of residence, age, and sex. Wealth was calculated with the standard method used by INEI, i.e., from quintile 1 as the poorest to quintile 5 as the richest, and was defined in ENDES in terms of assets or wealth in the surveyed homes, rather than in terms of income or consumption. In ENDES, data regarding the characteristics of the houses and the availability of certain consumable goods and services directly related to the socioeconomic level were collected. Each house was then given a score, and each resident was assigned a value for the house in which they live; in this way, population quintiles of wellbeing or wealth were created following the methodology reported by Rutstein and Johnson ( https://goo.gl/5fFFwz). The caregivers’ educational level was categorized into none/kindergarten, primary, secondary, and higher education. The area of residence was categorized into urban and rural. The natural regions were categorized as follows: the Lima Metropolitan area, rest of the coastline, highlands, and jungle. Age was divided into three ranges (namely 0–2, 3–5, and 6–11 years) according to Technical Health Norm data for the control of growth and development of male and female children aged <5 years, as published by the Ministry of Health of Peru ( https://goo.gl/4C3Tc2). The last variable was sex (male/female).

***Enabling factors.*** The type of health insurance was considered to be an enabling factor. The Peruvian health insurance system is structured as follows: 1) public Integral Health Insurance (i.e., SIS, initials in Spanish), which is the only public health insurance in Peru and provides health services to low-income earners via health centers in small communities; 2) Social Security Health Insurance (i.e., EsSalud), which provides health services to employed workers and those depending on that worker, with centers typically located in provincial capitals; 3) Armed Forces and Police Health System for employees of these institutions and those who depend on them; and 4) private insurance for individuals who are able to pay insurance. Since the creation of SIS and the Law for Universal Insurance, 66% of the population receives care in public health centers that are managed by the Ministry of Health ^16^. On the basis of these subsystems, we divided the enabling factor into whether participants had no insurance, SIS insurance, EsSalud insurance, Armed Forces and Police Health System insurance, or private insurance.

### Data analysis

The ENDES database was downloaded ( http://iinei.inei.gob.pe/microdatos/) and imported to the Stata statistical software v14.1 (Stata Corporation, College Station, Texas, USA). The sampling patterns were specified on the basis of the strata, expansion, and design factors using the *svy* command. Categorical variables were described using absolute frequencies, and weighted proportions with 95% confidence intervals were estimated. Because of the high prevalence of access to oral health services, Poisson generalized linear models were used with robust variance, and the log link function was used with the dependent variable. For the adjusted model, we used variables that showed a minimal association (p < 0.2) with access to oral health services, and prevalence ratios (PRs) were reported with 95% confidence intervals, with the assumption that p values of <0.05 were statically significant.

### Ethical considerations

This study did not require the approval of our ethics committee because it only involved analysis of secondary data obtained from a public and freely accessible source which does not require the identification of participants. That retains participant anonymity.

## Results

**Figure 1.**
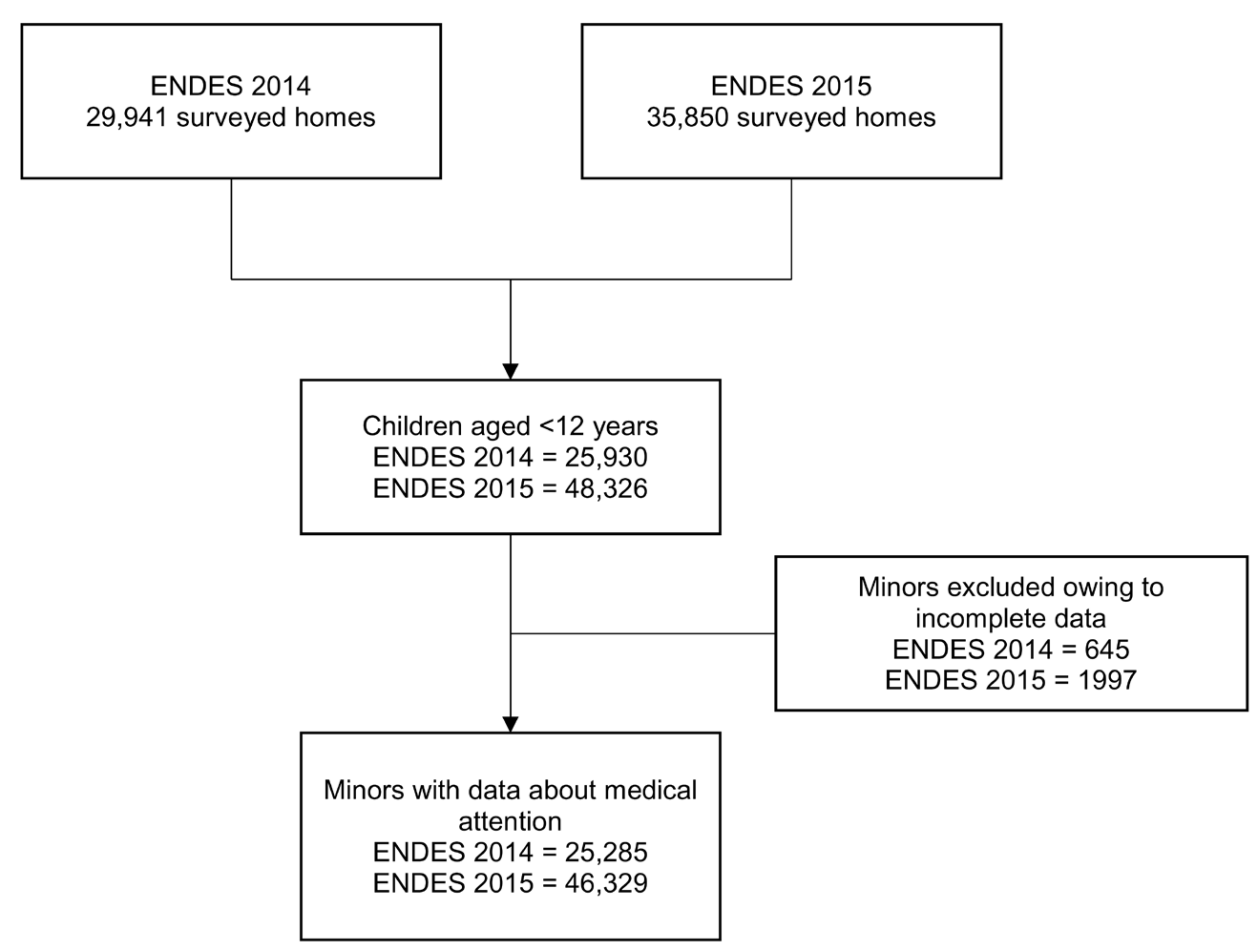
Elaboration of the selected sample for ENDES 2014–2015. The figure shows: number of surveyed homes, number of children aged <12 years, exclusions and final sample.

The flow chart for the study inclusion is shown in Figure 1, and the sociodemographic characteristics of the 71,614 included children are summarized in Table 1. In total, 51% were males, 56% were aged <5 years, and 44% were aged 6–11 years. In addition, 62.6% lived in urban areas and 35.6% lived in highlands. The most common type of health insurance cover was SIS (57.8%), whereas the least common type was private insurance (0.5%). Most children belonged to the first quintile of wealth (31.6%, 26.9%, 18.93%, 13.62%, and 9.75% in the first, second, third, fourth, and fifth quintiles, respectively). The most common education level of caregivers was high school (43.02%) and the least common was none/kindergarten (3.8%). The most frequently spoken language by caregivers was Spanish (88.4%), but a sizable portion spoke other languages (11.6%).

**Table 1.** Characteristics of Peruvian children aged <12 years who accessed dental health services (ENDES 2014–2015; N = 71,614).

Characteristic	No.	%
Age, years		
Mean (SD)	5.09 (3.42)	-
Sex		
Male	36,491	50.96
Female	35,123	49.04
Age group, years		
0–2	20,563	28.71
3–5	19,482	27.20
6–11	31,569	44.08
Area of residence		
Urban	44,818	62.58
Rural	26,796	37.42
Natural Region of Residency		
Lima Metropolitan area	7448	10.40
Rest of the coastline	19,631	27.41
Highlands	25,509	35.62
Jungle	19,026	26.57
Type of health insurance		
No insurance	14,433	20.15
Armed Forces	507	0.71
Integral Health Insurance (SIS)	41,370	57.77
Social Security (EsSalud)	14,964	20.90
Private	340	0.47
Quintile of wealth		
First quintile	22,629	31.60
Second quintile	18,683	26.09
Third quintile	13,560	18.93
Fourth quintile	9757	13.62
Fifth quintile	6985	9.75
Caregivers’ educational level		
None/kindergarten	2674	3.75
Primary	22,254	31.21
Secondary	30,673	43.02
Higher	15,703	22.02
Caregivers’ language		
Spanish	60,012	88.44
Other	7844	11.56

SD, standard deviation.

The univariate analysis revealed that all independent variables, except sex and primary educational level, were statistically significant. After adjusting the model for all variables, we found that sex, geographical location, and language spoken were insignificant ( Table 2). In this model, the probability of access to oral health services increased with age, with the probability being three times higher in the group aged 6–12 years than in the group aged 0–2 years (95% CI, 2.91–3.23). Moreover, children living in highlands had a higher likelihood of access to oral health services (PR, 1.06; 95% CI, 1.01–1.11) than those who living in the Lima Metropolitan area, rest of the coastline area, and jungle, with children in the latter two groups also showing lower probabilities of access to oral health services than those living in the Lima Metropolitan area. Another relevant finding was that children from the first quintile of wealth had lower probabilities of access to oral health services than those from the other quintiles. The probability of access to oral health services tended to increase as the quintile of wealth increased, with the probability being 1.61 times higher in the fifth quintile (95% CI, 1.48–1.74) ( Table 2). Caregivers’ higher educational level was also associated with a higher probability of access to oral health services, with the highest probabilities corresponding to children whose caregivers had a higher education level (PR, 1.63; 95% CI, 1.47–1.82). In contrast, the adjusted model indicated that the language spoken by caregivers was statistically associated with the access to oral health services ( Table 2).

**Table 2.** Factors associated with access to dental health services in Peru (ENDES 2014–2015).

Characteristic	Minors who had access to dental health services within the previous 6 months	Crude model			Adjusted model *		
	(%)	PR	(95% CI)	p	RP	(95% CI)	p
Sex							
Male	30.0	1	-	-	1	-	-
Female	29.9	1.00	(0.97–1.03)	0.949	0.99	(0.97–1.03)	0.763
Age group, years							
0–2	12.9	1	-	-	1	-	-
3–5	28.9	2.22	(2.09–2.35)	<0.001	2.25	(2.12–2.38)	<0.001
6–12	38.5	2.94	(2.79–3.10)	<0.001	3.06	(2.91–3.23)	<0.001
Area of residence							
Urban	34.4	1	-	-	1	-	-
Rural	23.0	0.67	(0.65–0.69)	<0.001	0.99	(0.95–1.04)	0.705
Natural Region of residency							
Lima Metropolitan area	39.4	1	-	-	1	-	-
Rest of the coastline	29.2	0.74	(0.71–0.77)	<0.001	0.85	(0.81–0.89)	<0.001
Highlands	30.3	0.76	(0.73–0.80)	<0.001	1.06	(1.01–1.11)	0.020
Jungle	21.1	0.53	(0.51–0.56)	<0.001	0.79	(0.75–0.84)	<0.001
Type of health insurance							
No insurance	23.1	1	-	-	1	-	-
Armed Forces	38.9	1.69	(1.43–1.99)	<0.001	1.21	(1.02–1.43)	0.030
Integral Health Insurance (SIS)	27.8	1.21	(1.15–1.27)	<0.001	1.40	(1.33–1.47)	<0.001
Social Security	41.7	1.81	(1.72–1.90)	<0.001	1.44	(1.37–1.52)	<0.001
Private	40.7	1.74	(1.43–2.11)	<0.001	1.32	(1.09–1.59)	0.004
Quintile of wealth							
First quintile	20.6	1	-	-	1	-	-
Second quintile	28.2	1.35	(1.29–1.41)	<0.001	1.26	(1.20–1.33)	<0.001
Third quintile	34.4	1.65	(1.58–1.73)	<0.001	1.46	(1.37–1.56)	<0.001
Fourth quintile	37.2	1.79	(1.70–1.88)	<0.001	1.51	(1.40–1.62)	<0.001
Fifth quintile	43.8	2.11	(2.01–2.22)	<0.001	1.61	(1.48–1.74)	<0.001
Caregivers’ educational level							
None/kindergarten	20.5	1	-	-	1	-	-
Primary	22.5	1.12	(1.02–1.23)	0.021	1.15	(1.04–1.27)	0.007
Secondary	31.4	1.56	(1.42–1.71)	<0.001	1.45	(1.31–1.60)	<0.001
Higher	40.3	2.00	(1.82–2.19)	<0.001	1.63	(1.47–1.82)	<0.001
Caregivers’ language							
Spanish	30.8	1	-	-	1	-	-
Others	22.8	0.74	(0.70–0.78)	<0.001	0.98	(0.93–1.04)	0.502

Poisson regression models were used with robust variance. PR, prevalence ratio. *Adjusted by all the variables shown in the column.

## Discussion

In this study, we identified several factors that determined access to oral health services. We showed that wealth index, caregivers’ education level, natural region of residence, and age were significant predisposing factors, whereas the type of health insurance was a significant enabling factor. However, we did not assess need factors as determinants of access.

As expected, wealth influenced the probability of access to oral health services among Peruvian children aged <12 years, which is compatible with the findings in studies of other Latin American countries and other countries worldwide. It is globally established that wealth plays a key role in oral health services, from access as well as prevention of oral disease for maintenance of better hygiene habits ^17– 19^. However, universal health insurance, aimed at covering the population living in poverty and extreme poverty and reaching 66% of the population in 2011, should improve the negative impact of the lack of resources on access to health services ^20^. Although this does not appear to be the case universally, programs such as JUNTOS, devised to encourage the Peruvian population to join health programs, appear to be achieving good results in some poor regions in Peru ( http://goo.gl/J7LlFJ). Nevertheless, the situation is more precarious when viewed at the national level, with evidence that the potential access to oral health services in Peru is incongruent with the actual access. Although numerous factors likely account for these dissimilar results, they probably include the limited portfolio of dental services in some health subsystems.

The primary caregivers’ lack of knowledge about oral health could have a negative impact on the oral health of a child ^21^. Consistent with our results, such knowledge correlates to a person’s educational level ^22^. We showed that children whose caregivers had a higher education level were more likely to have accessed oral health services within the 6 months before the survey. In Colombia, where most caregivers had a low educational level, 72.5% of the children had never been examined by an odontologist, although 97.5% had access to a general health social security service ^23^. Another study among children aged 7, 9, and 12 years in Lithuania showed that children whose parents had high educational levels were more likely to be informed and to receive information regarding oral hygiene than those whose parents had a low educational level (73.5% vs. 58.0; p < 0.001). Likewise, children whose parents had high educational levels and sufficient family wealth were 1.34 (95% CI, 1.05–1.71) and 1.71 (95% CI, 1.35–2.16) times more likely to visit a dentist for preventive revision than those whose parents had low educational levels and wealth ^18^.

We identified that the region with the highest probability of access to oral health services for children was the Peruvian highlands. These findings reinforce those of another study ^24^ where the frequency of access to oral health services was higher in the Peruvian highlands than in coastal or jungle regions. This result corresponds to the regions where the JUNTOS program has been in place the longest according to its performance report ( http://goo.gl/9psqai). In these cases, access may be improved because the JUNTOS program provides economic incentives to expectant mothers and those aged <19 years if their family are in extreme poverty. In exchange, participants must commit to using preventive health services, attending growth and development checkups, and engaging in child and adolescent education.

There was a clear correlation between a child’s age and his or her access to oral health services, with access being the highest among children aged >6 years. This may be because of the greater influence of caregivers’ beliefs, habits, and knowledge regarding oral healthcare for children aged <6 years ^4, 25^. This situation is equally apparent in other countries such as India, where 59.08% of children first visited a dentist at the age of 6–12 years, with toothaches typically being the main reason for visitation the dentist (42.04%) ^26^. Moreover, only 8.52% and 32.40% of children in that study had their first dental visit at the ages 0–3 and 3–6 years, respectively ^26^. Such results contradict the current recommendations that state that the first dental visit should occur during the first year of the child.

During the analysis of enabling factors, we found that access to oral health services was mediated by the type of health insurance, which is an interesting result because according to the theory of Andersen (the author of the model used in this study) ^27^, a health system is inequitable when enabling factors influence the effective access to oral health services; it is the predisposing and need factors that facilitate an equitable health system. This reinforces the idea that universal health insurance does not, by itself, determine access to health services ^28, 29^. Therefore, it is necessary to consider other factors that determine access and to develop social programs that focus on ensuring effective health service use and not merely on achieving a greater coverage ^30^. We must prevent the development of the “inverse care law,” by which people with the greatest need are the least served, and address the concern that health services exposed to market forces have increased inequality ^31^. Although we could not find a study that evaluated the factors that determined access to oral health services for the age group we analyzed, a study ^32^ conducted in Chile, which included all age groups, found that the probability of not receiving dental care was highest for people from lower socioeconomic groups, indigenous ethnic groups, and rural areas, as well as those receiving public health insurance. This result is consistent with the results of the current study.

A principal limitation of this study is that its cross-sectional design precludes establishing causal associations. This is compounded by the use of secondary data from ENDES, which limits the precision and accuracy of the collected data. Another limitation, albeit minor, is that the survey may have included two or more siblings, which prevents us from claiming the independence of data regarding the parent or caregiver of the child. It was also problematic that ENDES lacked suitable variables for assessing the need for access to oral health services and that this may be an area that needs to be changed in future studies of ENDES. The American Association of Pediatric Dentistry recommends semi-annual visits to the dentist, which supports our argument that all participants in the study required greater access to an oral health service.

Despite the study limitations, it should be noted that ENDES was a robustly performed, national study that required all participating surveyors to undergo training and standardization. The data generated in the current study may therefore be relevant to strengthening and improving oral health programs in Peru among children aged <12 years because, to date, there have been few studies that assessed the factors associated with access to oral health services. However, in the future, we recommend prospective studies that use primary data to collect information regarding access to oral health services and its determinants in Peru.

In conclusion, we showed that wealth index, education level, natural region of residence, age, and type of health insurance determine access to oral health services among children aged <12 years in Peru. When analyzing these results in the light of Andersen and Col’s theoretical model, we concluded that there was inequitable access to oral health services in Peru, which we consider was most likely because of the fragmented and inequitable healthcare system. Therefore, we make several recommendations to public health policy makers that may improve the current situations. First, it is essential that the gaps in oral health treatments offered through each subsystem be closed so that the population has truly equal opportunities. Second, the strategies used to promote and prevent oral health should focus on improving knowledge and demystifying oral health among parents and children, targeting those with the lowest probabilities of access to oral health services (i.e., pre-school children, those in displaced populations, and those whose parents have low educational levels). Third, the reach of social programs such as JUNTOS should be increased to cover regions where access to oral health services is not optimized because such programs appear to be effective in improving access, specifically in regions where the poverty levels are high. We hope that decision makers in Peru consider these recommendations and attempt to improve access to oral health services among children aged <12 years. By targeting this age group, we believe that better oral health outcomes can be achieved in the long term.

## Data availability

The data referenced by this article are under copyright with the following copyright statement: Copyright: © 2017 Azañedo D et al.

Data associated with the article are available under the terms of the Creative Commons Zero "No rights reserved" data waiver (CC0 1.0 Public domain dedication). 

Dataset 1: ENDES 2014–2015

The data was obtained from Instituto Nacional de Estadística e Informática ( http://iinei.inei.gob.pe/microdatos/) and merged by the authors. 10.5256/f1000research.12474.d176748 ^33^
