# Supplementary material for: Factors determining access to oral health services among children aged less than 12 years in Peru
Source: F1000Res. 2017 Sep 12;6:1680. [Version 1] doi: 10.12688/f1000research.12474.1 (PMC5820620; doi:10.12688/f1000research.12474.1)
Supplement: Peer review history

# Peer review: Referee response for version 1

**DOI:** 10.5256/f1000research.13507.r30717

**Published:** 2018-02-19

**Authors:** Efigênia Ferreira e Ferreira

**Affiliations:** 1 Dental School, Federal University of Minas Gerais, Belo Horizonte, Brazil

I appreciate the opportunity to read this manuscript. It is a population-based study with a well-planned and scientifically rigorous methodology, about access to dental care (dependent variable) for children <12 years old in Peru. It discusses some independent variables, and exposes the absence of dental care, especially for younger children (<5 years old).

 It uses the theoretical model of Andersen (1995) and points as factors associated with access: age group years, natural region of residency, type of health insurance, quintile of wealth and caregivers' educational level.

 In my point of view, the manuscript has quality, is a relevant study and is suitable for this journal.

 Only one suggestion. On page 5, at the end of the first paragraph the authors report: In contrast, the adjusted model indicated that the language spoken by caregivers was statistically associated with the access to oral health services (Table 2). However, this association was not observed in the adjusted model and was not part of the study's findings. I suggest that this statement should be revised.

I have read this submission. I believe that I have an appropriate level of expertise to confirm that it is of an acceptable scientific standard.

# Peer review: Referee response for version 1

**DOI:** 10.5256/f1000research.13507.r28106

**Published:** 2017-11-17

**Authors:** Sonia Constanza Concha Sánchez

**Affiliations:** 1 School of Dentistry, Universidad Santo Tomás, Bucaramanga, Colombia

I would recommend some modifications in the results section. In the second paragraph it should be reported that "The univariate analysis revealed that all independent variables, except sex, were statistically significant”. After adjusting the model for all variables, we found that sex, geographical location, and language spoken were no significant…”

 On the other hand, in the last paragraph I suggest referring ... "In contrast, the adjusted model indicated that the language spoken by caregivers was not statistically associated with the access to oral health services,

I have read this submission. I believe that I have an appropriate level of expertise to confirm that it is of an acceptable scientific standard.

# Peer review: Referee response for version 1

**DOI:** 10.5256/f1000research.13507.r27559

**Published:** 2017-11-16

**Authors:** Ana Flávia Granville-Garcia

**Affiliations:** 1 Postgraduate Program in Dentistry, State University of Paraíba (UEPB), Campina Grande, Brazil

Title: Suitable for what you propose.

 Summary: Insert level of significance ..

 Introduction: I inserted data from other countries, to situate the reader on the subject in the world and not only in the country being studied.

 Method: The study used retrospective data with a sample of considerable size. In these data are not registered the experience of caries? It would be interesting to see the association between caries experience and going to the dentist. Why not use your dental history data for more than six months. Did the trip to the dentist in the last six months, in fact, reflect the problem?

 Results: well described.

 Discussion: Adequate and associated variables were discussed. I suggest a more consistent conclusion reporting the strategies to be carried out more clearly. In addition, I suggest that perhaps the authors could talk a bit about literacy in oral health that is problematic that comes before all the problems cited in the article.

I have read this submission. I believe that I have an appropriate level of expertise to confirm that it is of an acceptable scientific standard, however I have significant reservations, as outlined above.
